# Acute effects of a 60-min time trial on power-related parameters in trained endurance runners

**DOI:** 10.1186/s13102-022-00538-7

**Published:** 2022-07-24

**Authors:** Antonio Cartón-Llorente, Luis E. Roche-Seruendo, Elena Mainer-Pardos, Hadi Nobari, Alberto Rubio-Peirotén, Diego Jaén-Carrillo, Felipe García-Pinillos

**Affiliations:** 1 Health Sciences Faculty, Universidad San Jorge, Autovia A23 km 299, 50830 Villanueva de Gállego, Saragossa, Spain; 2 Faculty of Sport Sciences, University of Extremadura, 10003 Cáceres, Spain; 3 Department of Exercise Physiology, Faculty of Educational Sciences and Psychology, University of Mohaghegh Ardabili, Ardabil, 56199-11367 Iran; 4 Department of Motor Performance, Faculty of Physical Education and Mountain Sports, Transilvania University of Braşov, 500068 Braşov, Romania; 5 Sports Scientist, Sepahan Football Club, Esfahān, 81887-78473 Iran; 6 Department of Sports and Physical Education, University of Granada, Granada, Spain; 7 Department of Physical Education, Sports and Recreation, Universidad de La Frontera, Temuco, Chile

**Keywords:** Fatigue, Athletic performance, Technology assessment, Running

## Abstract

**Background:**

The advent of power meters for running has raised the interest of athletes and coaches in new ways of assessing changes in running performance. The aim of this study is to determine the changes in power-related variables during and after a strenuous endurance running time trial.

**Methods:**

Twenty-one healthy male endurance runners, with a personal record of 37.2 ± 1.2 min in a 10-km race, completed a 1-h run on a motorized treadmill trying to cover as much distance as they could. Before and after the time trial the athletes were asked to perform a 3-min run at 12 km h^−1^. Normalized mean power output, step frequency, form power and running effectiveness were calculated using the Stryd™ power meter. Heart rate (HR) and rating of perceived exertion (RPE) were monitored, and data averaged every 5 min.

**Results:**

Despite high levels of exhaustion were reached during the time trial (HRpeak = 176.5 ± 9.8 bpm; RPE = 19.2 ± 0.8), the repeated measures ANOVA resulted in no significant differences (*p* ≥ 0.05), between each pair of periods for any of the power-related variables. The pairwise comparison (*T* test) between the non-fatigued and fatigued constant 3-min runs showed an increase in step frequency (*p* = 0.012) and a decrease in form power (*p* < 0.001) under fatigue conditions, with no meaningful changes in normalized mean power output and running effectiveness.

**Conclusions:**

Trained athletes are able to maintain power output and running effectiveness during a high demanding extended run. However, they preferred to reduce the intensity of vertical impacts under fatigue conditions by increasing their step frequency.

## Introduction

Fatigue is a psychophysical state of lack of energy that causes an acute inability to produce or maintain a desired power output. In endurance sports it leads athletes to make either conscious or unconscious decisions to modulate intensity or terminate the effort being hypothesized that in running activities these modulations reflect an altered neuromuscular function and reduced leg stiffness properties [1]. Furthermore, sustaining suboptimal lower limb mechanics over a prolonged repetitive activity such as endurance running is related with an increased risk of suffering overuse musculoskeletal injuries [2, 3].

However, the specific changes in running technique due to fatigue are not clearly stated to date and seem to depend on the nature of the fatiguing bout of exercise, the parameters assessed and the athlete’s level of performance [4, 5]. In this direction, the effects of fatigue on running economy have been studied in order to interpret performance impairments [6–8]. Running economy is defined as the energy demand for a given velocity of submaximal running [9], this is the ratio between velocity, as the outer expression of the energy applied, and oxygen uptake (VO_2_), as the metabolic cost of maintaining this velocity. Of note, running economy is considered one of the best indicators of how good an athlete’s technique is, and it was found to better predict performance in trained runners than maximal oxygen uptake (VO_2max_) alone [10].

1 $$Running\,economy = \frac{{{\text{metabolic}}\,{\text{power}}\,{\text{(mlO2/kg)}}}}{{{\text{Velocity}}\,({\text{km}}/{\text{h}})}}$$

Unfortunately, the acceptance of velocity as an outer expression of the effort’s intensity under in-field conditions results imprecise. Several external conditions such as wind, slope or terrain, might unpair metabolic cost for a given velocity, thus, its use could lead to misinterpretation of the actual exercise stress. In this context, the need for a more objective workload variable gave rise to the era of mechanical power assessment.

Mechanical power refers basically to the product of force and velocity. In cycling, strain gauges integrated in the crank or pedals directly assess the force applied and the angular velocity of these components, thus, power output is calculated multiplying the torque applied and the cycling cadence. As a result, wearable power meters became widely used in cycling because they aid decision making related with cycling technique, position and equipment [11]. Furthermore, power output demonstrated to be more reliable and sensitive than HR or velocity to address minimum variations in exercise intensity [12], thus, it became a key metric to guide training and racing strategies in the sport.

On the other hand, running mechanical power can be quantified using a treadmill with a force plate integrated which reflects ground reaction forces in the 3-axis at any given velocity, but such settings are costly, thus, some commercial companies have recently developed wearable power meters in a more practical approach to in-field running power assessment.

Novel running power meters estimate the force applied by the athletes derived from anthropometric measures (height and body mass) and spatiotemporal parameters (velocity, step rate and ground contact time). As a result, running power output can be calculated combining GPS technology and the 3-axis accelerometers and gyroscopes contained in small Inertial Measurement Units (IMUs) [13, 14].

Although the specific calculation algorithms still undisclosed by the companies and validity of the data obtained with these wearable devices has not been yet compared with the ‘Gold Standard’ (i.e., a force-plate-instrumented treadmill or a long force platform system), a recent study [15] assessed their concurrent validity with metabolic demands (i.e., VO2) showing consistent results for some of these novel devices (r > 0.9).

This approach gave rise to the appliance of power-related metrics for an in-field running technique evaluation. Of note, Stryd™ power meter divides instant power output into its horizontal and vertical components. Thus, form power refers to the power needed to bounce perpendicularly against the ground (or “running in place power”) whereas power account for the whole power output needed to push the body forward. In addition, running effectiveness is defined as the ratio between speed (m/s) and normalized power output (W/kg). It expresses the ability of an athlete to translate work rate (i.e., power) into speed. Knowing an athlete’s individual running effectiveness may help to understand the relationship between power and fatigue and establish’fatigue thresholds’ to distinguish between physiologically steady intensities and non-sustainable efforts [16].

2 $$Running\,efficiency = \frac{{{\text{Velocity}}\,({\text{m}}/{\text{s}})}}{{{\text{Normalized}}\,{\text{mechanical}}\,{\text{power}}\,({\text{W}}/{\text{kg}})}}$$

Noteworthy, Burnley and Jones [17] investigated the duration-power relationship describing three intensity domains (i.e., power zones) with different physiological events explaining the onset of fatigue for each of them. While moderate-intensity exercise (i.e., below aerobic threshold) can be sustained for more than three hours and fatigue appears to have its origin in the central nervous system, a severe-intensity activity (i.e., above Critical Power) is only tolerable up to 40 min and metabolite-mediated processes seem to trigger the subsequent peripheral fatigue. Amidst these power domains, the inability to endure heavy-intensity tasks (i.e., above lactate threshold but below critical power) is explained by a combination of central and peripheral physiological phenomenon. These physiological boundaries highly depend upon the athlete aerobic profile and, as stated previously [17], need to be set on an individual basis.

Several studies investigated the role of fatigue in running after moderate-intensity (e.g., ultramarathon distance) [18] and severe-intensity domains (e.g., high intensity interval training) [19], whereas studies regarding the heavy-intensity zone are mainly focused on marathon distance runs [3]. Actually, not many studies focused on biomechanical changes due to fatigue in the upper limit of the heavy-intensity domain [20], and only a few [21–23] assessed running kinematics and running economy after a one-hour fatiguing protocol.

Of note, there is some controversy about the effects of fatigue on running performance, as fatigue has been shown to affect differently depending on the metrics evaluated. While a few studies found no significant differences in step frequency [21] gait variability [4] and running technique [24, 25], other works revealed changes in running economy [6], energy cost [7] and propulsive forces [21, 26] due to fatigue. Thus, in the current work it has been hypothesized that a one-hour near to maximal time trial induces changes in power-related running metrics as the end of the test approached. Therefore, the aim of the current study is to evaluate the changes in normalized mean power output, step frequency, form power and running effectiveness during and after a strenuous one-hour time trial in trained endurance runners.

## Materials and methods

### Participants

A sample of trained male endurance runners was recruited from local running and triathlon clubs by sending the information to the coaches and then contacting the participants by email. The sample was selected by convenience to participate in this cross-sectional study. Participants met the inclusion criteria: (1) older than 18 years old, (2) able to run 10 km in < 40 min (i.e., 37.2 ± 1.2 min), (3) training on a treadmill at least once per week, (4) free from injury (points 3 and 4 refer to the six months preceding the study). After receiving information on the objectives and procedures of the study, participants signed an informed consent form, which complied with the ethical standards of the World Medical Association’s Declaration of Helsinki (2013). The study was approved by the local ethics committee.

### Procedures

Tests were individually scheduled on a particular day between March and July 2019. Participants were encouraged to maintain their normal dietary pattern and asked to refrain from severe physical activity for 48 h before the test, and from eating and consuming stimulants or ergogenic aids at least three hours before the study begins.

All subjects were asked to complete a near maximal one-hour run on a motorized treadmill (HP cosmos Pulsar 4P; HP cosmos Sports and Medical, Gmbh, Nußdorf, Germany) inclined 1% to reflect outdoor running conditions [27] and wearing their usual competition shoes to avoid technical changes in their performance. Additionally, a 3-min run at 12 km/h before (i.e., non-fatigued) and immediately after (i.e., fatigued) the one-hour time trial, was also recorded for comparative purposes.

### Materials and testing

At the beginning of the testing session, body height (m) and body mass (kg) were measured using a precision stadiometer and weighing scale (SECA 222 and 634, respectively, SECA Corp., Hamburg, Germany). Additionally, all the athletes were instructed on the use of the 6–20 Borg’s rate of perceived exertion scale (RPE) [28, 29].

For all tests, temperature and humidity were kept between 18 and 20 °C and 50–60% respectively, using a wireless weather station (Ea2 LABS DE903), and ventilation was assured with two industrial fans located laterally at two meters distance from both sides of the treadmill. Fluid intake was ad libitum during the entire protocol. Participants received verbal encouragement from the same investigator to complete as much distance as they can, and slight velocity variations were permitted throughout the 60-min run.

Considering that accommodation to running on a treadmill typically occurs in ~ 6–8 min [30], an 8-min standardized warming up protocol (i.e., 4-min at self-selected velocity and 4-min near their expected speed for the test) was included. After the accommodation period and during the three parts of the test (i.e., 3-min non-fatigued, 1-h time trial and 3-min fatigued) HR was monitored continuously using a portable HR monitor (Polar, FS2c, Kempele, Finland), and RPE was assessed every 5 min until the end of the time trial. Mean power output (normalized by body mass), step frequency, form power and running effectiveness were calculated using the Stryd™ power meter (Stryd Power meter, Stryd Inc. Boulder CO, USA) attached on the upper part of the running shoes. This sensor provides accurate kinematic [31, 32] and consistent power output metrics [15]. Data from Stryd™ power meter were obtained into the fit file via the manufacturer’s website (https://www.stryd.com/powercenter/analysis) and analyzed using a free software (Golden Cheetah, version 3.4) being exported, thereafter, as.csv file into Excel® (2016, Microsoft, Inc., Redmond WA).

Data were recorded and averaged for the subsequent analyses as follows:

- 3 min non-fatigued.
- Every 5 min during the 1-h time trial.
- 3 min fatigued.

### Statistical analysis

Descriptive statistics are represented as mean (standard deviation, SD). The normal distribution of data and homogeneity of variances were confirmed through the Shapiro–Wilk and Levene’s tests, respectively (*p* > 0.05). A repeated measure analysis of variance (ANOVA) was conducted for running velocity, HR, RPE, normalized mean power output, step frequency, form power and running effectiveness to examine differences between the different time periods during the 60-min time trial (i.e., 0–5 min, 5–10 min, 10–15 min, 15–20 min, 20–25 min, 25–30 min, 30–35 min, 35–40 min, 40–45 min, 45–50 min, 50–55 min and 55–60 min). A Bonferroni post-hoc test was performed when needed. Additionally, pairwise comparisons (i.e., *t* test) were also conducted for each power-related parameter in order to examine possible differences between non-fatigued vs. fatigued condition. The magnitude of the differences was interpreted using the Cohen’s effect size (ES) (between-group differences) [33]. Effect sizes are reported as: trivial (< 0.2), small (0.2–0.49), medium (0.5–0.79), and large (≥ 0.8) [33]. The level of significance used was *p* < 0.05. Data analysis was performed using the SPSS (version 21, SPSS Inc., Chicago, Ill).

## Results

Twenty-one trained male endurance runners (age: 35.5 ± 7.3 years; height: 1.76 ± 0.04 m; body mass: 71.1 ± 5.9 kg; 42 ± 16 km/week; > 2 years training experience) participated in this cross-sectional study.

Markers of external (i.e., running velocity) and internal load (i.e., HR and RPE) are shown in the Table 1. Data are presented in 5-min intervals during the 60-min running protocol. The running velocity was constant during the time trial with no differences between time periods (*p* = 0.507), whereas the HR and RPE increased throughout the protocol with the repeated measures ANOVA showing significant differences (*p* < 0.001 for HR and *p* = 0.001 for RPE) and the Bonferroni post-hoc test reporting significant differences between each pair of periods.

**Table 1:** External and internal load indicators during a 60-min running time trial on treadmill considering 5-min time periods

Time periods	Running velocity (km h^−1^)	Heart rate (bpm)	RPE (6–20)
0–5 min	15.2 (0.5)	154.5 (9.3)	10.7 (3.2)
5–10 min	15.3 (0.5)	159.9 (9.8)	12.2 (2.5)
10–15 min	15.3 (0.5)	161.9 (9.9)	13.3 (2.4)
15–20 min	15.3 (0.5)	163.8 (9.9)	14.1 (2.2)
20–25 min	15.2 (0.5)	165.3 (9.7)	14.8 (2.1)
25–30 min	15.2 (0.6)	166.5 (9.5)	15.4 (1.9)
30–35 min	15.2 (0.6)	167.6 (9.4)	16.0 (1.6)
35–40 min	15.2 (0.6)	168.6 (9.5)	17.1 (1.5)
40–45 min	15.2 (0.6)	169.7 (9.5)	17.6 (1.5)
45–50 min	15.1 (0.6)	170.7 (9.6)	18.4 (1.3)
50–55 min	15.1 (0.7)	171.7 (9.9)	18.7 (1.3)
55–60 min	15.4 (0.8)	173.4 (9.9)	19.2 (0.8)
Average	15.2 (0.6)	166.2 (9.7)	–
Maximum	–	176.5 (9.8)	19.2 (0.8)
*p* value	0.507	< 0.001	0.001

*bpm* beats per minute, *RPE* rate of perceived exertion

**Fig. 1:**
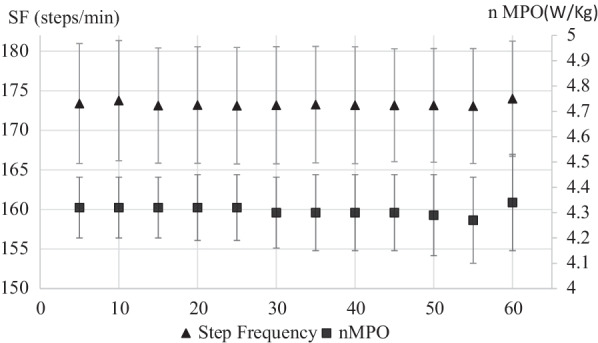
Step frequency and power output during a 60-min running protocol considering 5-min time periods. Triangles shows step frequency (steps/min) and squares shows power relative values (W/kg). *SF* step frequency, *nMPO* normalized-by body mass-mean power output

The Fig. 1 shows the dynamic of step frequency and power output during the 60-min time trial, considering the aforementioned time periods. No significant differences were found between intervals for step frequency (steps/min) nor for normalized power output (W/kg) (*p* = 0.067 and 0.244, respectively).

**Fig. 2:**
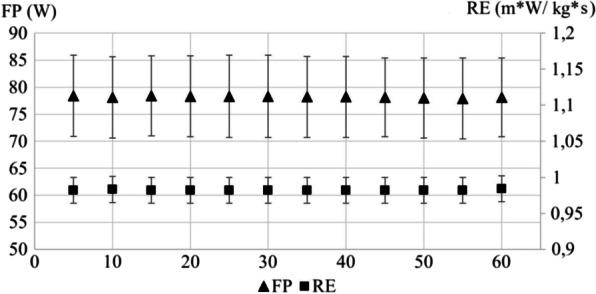
Power-related variables (i.e., form power [triangles] and running effectiveness [squares]) during a 60-min running protocol considering 5-min time periods. *FP* Form power, *RE* running effectiveness

The response of power-related variables (i.e., form power and running effectiveness) to the 60-min running time trial is shown in Fig. 2. No significant differences were found between the different time periods for any variable (form power, *p* = 0.268; running effectiveness, *p* = 0.067).

The results of the pairwise comparison (i.e., *t* test) between the non-fatigued and fatigued 3-min runs at 12 km/h are shown in Table 2. A moderate increase in step frequency (0.966 steps/min; *p* = 0.012; effect size = 0.602) and a large decrease in form power (− 2.093 W; *p* < 0.001; effect size = 1.068) were found under fatigue conditions, whereas normalized mean power output and running effectiveness reported minimum changes with small effect sizes (− 0.014 w/kg; effect size = 0.452), and 0.004 m*W/kg*s; effect size = 0.464, respectively).

**Table 2:** Pairwise comparisons (*t* test) between non-fatigued and fatigued 3-min run at 12 km/h, before and immediately after a 60-min running time trial

Variable	Non-fatigued	Fatigued	*p* value	ES (d)	Mean difference (CI)
Mean power output (W)	249.6 (19.5)	248 (7.1)	0.053	0.45	− 1.0 (− 2.1, 0.1)
Normalized mean power output (W/kg)	3.51 (0.07)	3.50 (0.08)	0.051	0.45	− 0.01 (− 0.03, 0.00)
Form power (W)	76.9 (7.3)	74.7 (7.1)	< 0.001	1.07	− 2.1 (− 3.0, − 1.2)
Running effectiveness (m*W/kg*s)	0.95 (0.02)	0.95 (0.02)	0.046	0.46	0.01 (0.00, 0.01)
Step frequency (steps/min)	165.0 (7.0)	166.9 (6.4)	0.012	0.60	1.0 (0.2, 1.7)

*ES* Cohen’s d effect size, *CI* confidence interval

## Discussion

The main aim of this study was to determine whether power-related variables and running effectiveness change over a one-hour time trial in trained endurance runners. The results highlight that despite the athletes reached high levels of exhaustion (HRpeak = 176.52 bpm; RPE = 19.24), they were able to maintain power output, step frequency, form power and running effectiveness throughout the one-hour time trial, and no significant changes were found in their running mechanical work. Additionally, the comparison between fatigued versus unfatigued condition, evaluated at a fixed comfortable running velocity of 12 km/h pre- and post-test, reported a slight increase in step frequency and a reduction in form power under fatigue conditions, with no meaningful changes for power output and running effectiveness.

### 60-min trial

Regarding the evaluation of power-related variables during the 60-min near-maximal running test, the present work found no differences in power output, step frequency, form power and running efficiency between any of the 5-min periods in which the data were averaged. Our results seem to be in line with previous works [4, 21, 24, 25] which find a self-optimization strategy in trained runners that allows them to complete long-lasting runs without significant performance impairments. Particularly, Hanley [24] noted that despite slight changes in contact and flight times, runners demonstrated their ability to maintain running technique stable throughout the race. Furthermore, Roelands [25] attribute this ‘pacing’ ability to a well-developed teleoanticipation mechanism, that is, an interplay between feedforward and feedback internal systems that enables the regulation of power output in order to better distribute energy resources throughout the run.

Conversely, various studies found a decrease in running economy [6], energy cost [7], and propulsive forces [21, 26] in running under fatigue conditions. In particular, Hunter & Smith [21], evaluated the effects of different step frequencies on the running economy of sixteen trained athletes during a constant velocity run of one hour (i.e., a similar but not equal condition to our study as we allowed little velocity changes throughout the run) finding a step frequency optimization at the beginning and the end of the protocol. Noteworthy, their results also showed an overall increment of ⁓3% by the last minutes of the run, but the authors acknowledge that considerable differences in individual athlete response to fatigue were detected, suggesting that the effect of fatigue on running economy might be subject-specific. Additionally, as mentioned above, the physiological mechanisms of fatigue depend upon the intensity and duration of the effort therefore the different fatiguing protocols used in these studies may explain this discrepancy.

Despite the existing controversy, the results here reported highlight the ability of trained athletes to keep their work rate and speed constant even when high levels of fatigue (e.g., RPE > 18) threaten to jeopardize their performance. As Lacour and Bourdin [7] pinpointed in a previous review, a variance of 20% of energy cost could be found between runners depending on body dimensions and level of performance, thus, the results shown in the present study might not be generalized to different training level athletes.

### Pre-post analysis

The comparison between the fatigued and unfatigued 3-min run at a fixed comfortable running velocity for those athletes (i.e., 12 km/h), revealed a small increase in step frequency (~ 1 step/min) and a reduction in form power (− 2.093 W), with negligible changes in normalized mean power output and running effectiveness under fatigue conditions.

Arguably, the impossibility to adapt the velocity during the 3-min run gave rise to the appearance of slight changes in power-related variables. In this scenario, Austin et al. [34] highlight the sensitivity of form power to changes in cadence, so that a slight increase in cadence might explain the lower power needed to counteract gravity vertically (i.e., lower form power). It is well known that running requires the generation of forces against the ground to propel the body upward and forward (i.e., external work), as well as forces on the various body segments to return them to their initial position at the beginning of the cycle (i.e., internal work). Under constant velocity conditions, an increase in step frequency would imply a reduction in external forces (i.e., against the ground), while internal forces to reposition the leg would increase [35]. Therefore, a possible explanation for the large increase in form power found under fatigued conditions (mean difference = − 2.093 W; confidence interval (− 2.986, −1.201) might be the need to minimize vertical impacts due to a lower limb reduced shock absorption capacity.

Nevertheless, it might be suggested that the trivial effect sizes obtained for the rest of power-related variables (i.e., normalized mean power output, running effectiveness) confirm the well-developed ability of trained endurance athletes to adapt themselves and maintain their mechanical effectiveness stable after a heavy-intensity run. Furthermore, the authors suggest that a 3-min run at 12 km/h is not such a challenging goal for trained runners, even after a one-hour time trial, thus the negligible differences observed in the pre-post analysis should be interpreted cautiously.

As first suggested by Roelands et al. [32] in their approach to the neurophysiological factors influencing ‘pacing’ ability of trained endurance runners, some volitional adjustments could also explain these findings. Unfortunately, the absence of previous research assessing the role of fatigue in these novel running metrics makes difficult to draw further conclusion in light of the current data.

Finally, some limitations should be taken into account. Despite the consistency in power-related parameters observed in the current work, previous studies [20–22] found differences in other kinematic and kinetic variables (e.g., leg stiffness, step variability, hip and knee angles) that might be connected to those. In this regard, it should be recognized that video recording would have allowed additional analysis of kinematic parameters during the protocol. However, as Winter et al. [5] addressed in their systematic review, consensus has not been reached yet and further evidence is needed to determine the relationship between fatigue and novel running metrics such as the ones presented hereabout.

It should also be noted that the whole protocol was performed on a treadmill, remaining unknown the repeatability of these results in overground running. However, a recent review by Van Hooren et al. [36] showed that both conditions are largely comparable when a long enough accommodation period is conducted before testing. Additionally, laboratory conditions allowed us to standardize other potential influencing variables such us temperature, humidity or wind effect.

Notwithstanding these limitations, the current study offers relevant insights into the impact of fatigue on power output, step frequency, form power and running effectiveness in endurance runners. Due to the large number of coaches and runners using the Stryd™ sensor, the information concerning the behavior of power-related metrics during endurance running is highly demanded. In addition, the very limited scientific evidence in this regard also puts in value the information here provided.

## Conclusions

To sum up, this work shows no changes in power output, step frequency, form power and running effectiveness in trained endurance runners during a strenuous one-hour time trial. From a practical standpoint, practitioners and coaches should know that despite the constant increase experienced in physiological parameters and the high level of exhaustion reached at the end of a 1-h endurance trial, trained athletes are capable to maintain their running effectiveness stable throughout the whole run, that is, their ability to translate power output into speed is not affected by such a demanding extended run.

## Data Availability

The datasets generated and analyzed for this study are available in the following OSF repository: https://osf.io/8d35z/.

## Abbreviations

HR: Heart rate
RPE: Rating of perceived exertion
